# Hallucinogen-Induced Persisting Perception Disorder: A Case Report

**DOI:** 10.7759/cureus.46262

**Published:** 2023-09-30

**Authors:** Javairia Ayyub, Sailaja Nandennagari, Dylan Edelbaum, Jennifer Agbo, Deenuka Nagendran, Luciano Tamayo

**Affiliations:** 1 Department of Medicine, Caribbean Medical University School of Medicine, Willemstad, CUW; 2 Department of Behavioral Health and Psychiatry, Avalon University School of Medicine, Willemstad, CUW; 3 Department of Behavioral Health and Psychiatry, Windsor University School of Medicine, Cayon, KNA; 4 Department of Behavioral Health and Psychiatry, Richmond Gabriel University, Arnos Vale, VCT; 5 Department of Behavioral Health and Psychiatry, Loretto Hospital, Chicago, USA

**Keywords:** hallucinogen persisting perception disorder, antiepileptic medications, benzodiazepines, alpha-2 adrenergic agonists, visual perception distortion, lysergic acid diethylamide

## Abstract

Hallucinogen-persisting perception disorder (HPPD), also known as acute hallucinogen-induced psychosis or informally known as "flashbacks," is an unusual condition experienced by patients due to the use of different hallucinogenic substances. Hallucinogen-persisting perception disorder causes many symptoms, predominantly persistent visual perception distortion instead of intermittent distortion. Although different hallucinogens could cause HPPD, lysergic acid diethylamide (LSD) and LSD-like properties seem to be the most common hallucinogens causing the symptoms. In our case report, the patient is a 28-year-old Caucasian male with a long psychiatric and social history of polysubstance use using LSD and cannabis. He started experiencing many of the classic symptoms of HPPD seven months after stopping LSD. The diagnosis is suspected by ruling out all other possible underlying causes with the help of several laboratory and imaging tests. Despite having an extensive psychiatric history of illnesses, the patient’s symptoms failed to improve with antipsychotics, confirming that the symptoms were not only due to mental illness. Although supposedly the first-line treatment for HPPD is the use of alpha-2 adrenergic drugs such as clonidine and benzodiazepines, we started to witness improvement in patient's symptoms with the use of lamotrigine, which is the gold standard in treating perceptual disturbance in time and space.

## Introduction

Hallucinogen-persisting perception disorder (HPPD) is characterized by persistent or recurrent perceptual abnormalities that are acutely experienced with hallucinogens. Several of the hallucinogens that have been linked to HPPD are lysergic acid diethylamide (LSD)-like substances, cannabis, methylenedioxymethamphetamine (MDMA), psilocybin, mescaline, and psychostimulants [1]. Hallucinogen-persisting perception disorder was initially described in 1954. However, it was first recognized as a clinical syndrome in 2000 and added to the revised Diagnostic and Statistical Manual of Mental Disorders (DSM-IV-TR) [1, 2]. According to the Fifth Version of the Diagnostic and Statistical Manual of Mental Disorders (DSM-5), HPPD is defined and diagnosed by three of the following criteria: a) HPPD is the recurrence of one or more of the hallucinogen-induced perceptual symptoms (such as geometric hallucinations, false perceptions of movements in the peripheral visual fields, flashes of color, intensified colors, trial images of moving objects, positive after images, haloes around objects, macropsia, and micropsia) after the cessation of hallucinogen use; b) criteria (a) symptoms create clinically considerable distress or impairment in social, professional, or other vital areas of functioning; c) the symptoms are not caused by other illnesses such as brain tumors, infections, or visual epilepsies as well as are not better explained by a mental illness such as delirium, dementia, or schizophrenia or hypnopompic hallucinations [1]. Apart from its diagnostic criteria, HPPD is more frequently identified in people with a history of mental illness or substance abuse. In addition, perceptual disturbances can be experienced by anyone months to years after complete cessation of hallucinogen; furthermore, it can occur in anyone even after one use of associated drugs [1, 2].

Hallucinogen-persisting perception disorder has two different types, and although both types have distinguishing symptoms from mild to severe, some of the overall symptoms are visual, such as micropsia, macropsia, floaters, fractals, monochromatic vision, acquired dyslexia, visual snow, and unique symptoms of visual perception. Apart from these symptoms, some of the non-visual symptoms experienced are recurrent synesthesia, dissociation, auras, depersonalization, and derealization. Along with these sensory problems and hallucinations, the patient may experience intense anxiety, which can develop into full-blown panic episodes [2]. Hallucinogen-persisting perception disorder type I is associated with reversible and short-lived “flashbacks" that are random in timing. Individuals tend to experience mild symptoms that do not affect their normal functioning. Its prognosis is usually good compared to type II. In contrast, HPPD type II is irreversible and causes long-term, persistent hallucinations. These symptoms are chronic and distressful, and the duration of the recurrent hallucinations might extend for months to years, “waxing and waning” in severity. The prognosis is worse for type II [2, 3]. It must be noted that the DSM-5 has not yet established a distinction between HPPD types I and II. While HPPD type II more closely resembles the DSM-5 criteria, HPPD type I is congruent with the diagnostic diagnosis provided by the International Classification of Disease, 10th Edition (ICD-10) [3].

We present a clinical case report of a patient who qualifies to have met with the diagnosis of HPPD despite having a past psychiatric history by ruling out all possible causes. Our patient developed and experienced some of the classic symptoms of HPPD seven months after stopping LSD.

## Case presentation

The patient is a 28-year-old Caucasian male who presented to the locked psychiatry unit due to a chief complaint of having serial killer fantasies and suicidal ideation with the plan to drink himself to death. The patient also complained of having chest pain and shortness of breath at the time of his admission. Upon asking the patient to explain his complaint, the patient stated, “I have become very corrupted and lost the poetry in my soul.” At the presentation, he was not receiving any treatments despite having a complex past psychiatric history of attention deficit disorder (ADD), anxiety, bipolar disorder, depression, post-traumatic stress disorder (PTSD), schizophrenia, and schizoaffective disorder. He was also not using drugs for recreational purposes, despite having a social history of polysubstance use since he was 14, including LSD and cannabis. The patient reported that he was regularly using LSD and occasionally using cannabis. However, there was a complete cessation of all substance use for seven months at his admission. Although he was not receiving any treatments for his health conditions at admission, he reported taking haloperidol, lithium, olanzapine, and risperidone in the past without recalling their doses. However, the patient could not tolerate the medicines and eventually stopped taking them. The patient's medical history was only significant for psychiatric illnesses.

The patient’s physical examination revealed a temperature of 35.9 degrees Celsius, a heart rate of 75 beats per minute, a blood pressure of 120/72 mm Hg, and a respiratory rate of 17 breaths per minute with an oxygen saturation of 100% on room air. According to the internal medicine team, his cardiac examination showed a regular rate and rhythm without any murmurs. His lungs were clear to auscultation bilaterally. The abdomen was soft and non-distended. According to the psychiatry team, the patient appeared disheveled but cooperative. Although anxious, depressed, and sad, his speech was normal in rate, rhythm, and tone. He had a normal standardized mini-mental exam (SSMSE). His mood was blunt, and his thoughts were delusional, illogical, and paranoid. He reported having auditory and visual hallucinations, “hearing voices in his head,” and “seeing people from the corner of his eyes.” Despite having acknowledged having suicidal thoughts about drinking himself to death, he has never actually carried out such plans in the past.

Laboratory analysis completed to rule out underlying etiologies included urodynamic testing (UDS) and blood alcohol level (BAL), which were within normal limits. Other significant tests included a complete blood count (CBC) panel, an electrolyte panel, a lipid panel, a liver panel, a renal panel, a thyroid panel, a blood glucose level, and a d-dimer level, all within normal limits.

The patient was admitted to the psychiatric facility for 11 days. The course of inpatient management started with the administration of 3 milligrams of Invega and 10 milligrams of Zolpidem. On day three, he reported feeling depressed and complained of other patients “messing with him psychologically.” He insisted that the medication is not working for him as he continues to have symptoms with little to no sleep. It was then decided to increase his dose of Invega to 6 grams and add 2 milligrams of lorazepam as needed (PRN) and 50 milligrams of trazodone. Later that night, the patient became moderately agitated, and 2 milligrams of lorazepam were administered. On the fourth day, the patient stated, "Objects appear and disappear from my site, and I see shadows" in his peripheral vision. He also said, "I see flashes of green and different colors that are hard to describe." Due to no improvements in his symptoms, 25 milligrams of lamotrigine were added. On the fifth day, the patient stated, "I see faces, and I am seeing dead philosophers," naming Alan Watts. He also reported intrusive thoughts in his head and believed his symptoms may be related to LSD. His regimen continued from the previous day; however, around that evening, the patient requested Haldol 5 milligrams and Ativan 2 milligrams orally for anxiety and scary visual hallucinations when he closed his eyes.

Despite the regimen, he continued to have ongoing symptoms. On the sixth day, the patient stated, “The space-time continuum is not constant. I can close my eyes for 45 seconds and open them, and I am in a different location than the one I am at. I see people are following and stalking me, and many big corporations are after me, like Google and especially Tesla.” He also reported that he was at the cemetery, sitting on a tombstone, contemplating his mortality. However, when he left the cemetery and walked about 45 minutes away from the graveyard, he somehow ended up in the exact location. Due to minimal improvement, lamotrigine was increased to 50 milligrams in the regimen. On the seventh day, the same regimen continued, and the patient reported improvements in having fewer intrusive thoughts and flashbacks. On the eighth day, the patient reported having no negative thoughts and feeling much better. He stated, “I am optimistic about the future and have better control over my thoughts.” He denied any perceptual distortion or hallucinations. On the ninth, tenth, and eleventh days, the same regimen continued with the improvement of an elevated mood of 6/10. The patient believed that the medications were finally working for him. He denied any intrusive thoughts or auditory or visual hallucinations. He was later discharged that day, following the same regimen.

**Figure 1:**
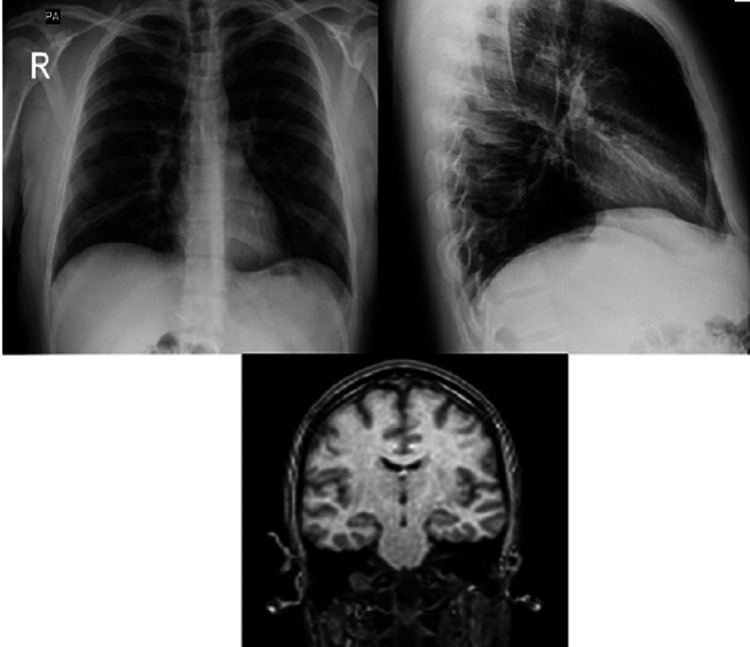
Anteroposterior and lateral views of chest X-ray and an MRI of the brain.

Assessments and imaging shown in Figure 1 were conducted during his course of inpatient management to rule out all possible underlying causes before diagnosing the patient with HPPD.

Imaging such as chest X-rays (CXR) of cardiopulmonary systems was performed to rule out underlying etiologies related to cardiopulmonary systems, such as infections (pneumonia). An electrocardiogram (ECG) was also performed to rule out cardiovascular etiologies. The ECG was standard, with a QT interval of 376 milliseconds. In contrast, brain imaging, such as an MRI of the brain without contrast, was used to rule out any cerebrovascular accidents (CVA), epilepsies, delirium, and space-occupying lesions in the brain. All tests were within normal limits. Before the patient’s hospitalization, he reported having an eye exam within normal limits.

Symptoms related to HPPD can have different possible causes, and therefore it is very crucial to rule out all possible underlying causes before diagnosing. Such was the course of action in our patient’s management described above. Some of the possible causes are anatomic brain lesions, brain infections (encephalitis), epilepsy, delirium, depersonalization, derealization, hallucinogen-induced psychotic mood and anxiety disorders, hypnopompic hallucinations, and schizophrenia [4].

Although there is no definitive treatment for HPPD, the supposed first-line treatment for HPPD is with alpha-2 adrenergic drugs such as clonidine and benzodiazepines such as alprazolam and clonazepam. Our patient's symptoms started to improve with an increasing dosage of 50 milligrams of lamotrigine, as shown in Table 1.

**Table 1:** Comparative tables highlighting effects of lamotrigine (pre-and post-treatment periods) DSM: Diagnostic and Statistical Manual of Mental Disorders

DSM IV/V Symptoms	Pre-Lamotrigine	Post-Lamotrigine
Altered Motion Perception	Present	Improved
Color Enhancement	Present	Improved
Flashes of Color	Present	Improved
Halos	Present	Improved
Trails or Tracers	Present	Improved
Visual Hallucinations	Present	Improved

## Discussion

Hallucinogens are among the oldest known classes of medications used for their ability to change perception and mood. They can be found in plants and fungi or can be synthetically manufactured. These drugs affect the body, producing sympathomimetic effects such as hypertension, mydriasis, and hypertension, and can frequently cause nausea and vomiting. Some of the most crucial side effects affect the mind, producing perceptual distortions. Individuals experience time- and space-related perceptual distortions. Time may seem to stand still while the shapes, colors, and hues of hallucinogens appear to shift and acquire new importance, eventually suffering from symptoms as mild as “flashbacks” and as severe and rare as hallucinogen-persisting perception distortion [5]. Although any hallucinogen can cause the symptoms, LSD seems to be the main trigger for developing HPPD. Individuals with a history of using hallucinogens are 4.0% to 4.5% more likely to develop HPPD, and there is no connection between the amount of drug consumed and the likelihood of HPPD [6].

One study suggests that one of the vital neurobiological theories for HPPD is that long-term disinhibition of visual processors results in central nervous system dysfunction from hallucinogens like LSD, leading to persistent hallucinations. Cortical serotonergic inhibitory interneurons, which are involved in the inhibitory neurotransmitter gamma-aminobutyric acid (GABA), may be destroyed or dysfunctional, leading to chronic disinhibition. This eventually disrupts the regular neurological processes that filter out superfluous stimuli for the brain. In addition, the long-term recurrence of hallucinations that can be observed following hallucinogen withdrawal may be caused by reverse tolerance or sensitization that develops after LSD exposure. It has also been suggested that the lateral geniculate nucleus (LGN) of the thalamus, which is crucial for visual processing, plays a role in the pathophysiology of HPPD on a macroscopic level [2]. According to another study, it is hypothesized that the excitotoxic degradation of inhibitory interneurons with serotonergic and GABAergic receptors on their cell bodies and terminals may be the pathophysiological cause of HPPD symptoms [7].

The results of all potential tests to rule out differential diagnoses, including assessments, imaging, laboratory analyses, and treatments, suggest that even though the patient had a complicated psychiatric history, the patient may have developed a hallucinogen-persisting perceptual issue.

The patient developed and reported several hallucinogen-induced perceptual symptoms for the first time after seven months of complete cessation of LSD. His symptoms affected enough of his social, professional, and other areas of functioning to be admitted to the hospital. However, Criteria C of the DSM-5 could not be met due to the patient’s history of schizophrenia. Nevertheless, one report claims that LSD-induced HPPD patients tend to exacerbate LSD-like panic and visual symptoms when prescribed risperidone in individuals with HPPD caused by LSD [7]. Another case reported a similar instance describing two LSD-abusing schizophrenia patients who experienced brief episodes of transitory visual disturbances shortly after starting their risperidone treatment. These visual disturbances mirrored what individuals have previously reported as “flashbacks” from using LSD. It is a brief phase during treatment that ceases to exist within six months of the treatment with antipsychotics [8].

While some of our patient’s symptoms may overlap with his psychiatric history, it is essential to note that although those diagnoses have been made in the past and he has been prescribed medications, he did not report episodes of said illnesses when he was off medications. A psychiatric illness such as schizophrenia involves persistent or recurrent psychosis that affects individuals' social and occupational functioning. The World Health Organization states it is among the top 10 diseases contributing to the global disease burden. It is one of the most debilitating and financially disastrous medical conditions [9]. Compared to the general population, individuals with schizophrenia consume hallucinogens, especially LSD, at higher rates, according to one report. In addition, one of the distinguishing factors among individuals with schizophrenia and HPPD is experiencing lower rates of negative symptoms, as these symptoms are linked to more serious functional deficiencies in schizophrenia. However, positive symptoms are similar in both conditions, making diagnosis difficult. A further important finding is that schizophrenia patients with comorbid HPPD have higher rates of having a "bad trip" than those without HPPD. This may suggest a link between specific immediate LSD effects and the chance of developing other pervasive LSD-related diseases. Furthermore, individuals who have schizophrenia and HPPD may not have HPPD-related perceptual symptom improvement [10]. In addition, another significant factor to emphasize is that individuals with HPPD know the unreal nature of their visual disturbances, which defines them as “pseudo-hallucinations” in contrast to those with conventional psychotic diseases, where individuals experiencing psychosis in psychiatric illnesses are unaware of the reality of their symptoms, believing that is their reality [11].

While research is still ongoing to find a permanent cure for HPPD, several medications that have been tested so far include benzodiazepines like alprazolam and clonazepam, as well as alpha-2 agonists like clonidine. However, our patient’s symptoms started to ameliorate with lamotrigine. Although several studies have reported HPPD symptom improvement with lamotrigine, one study highlights a patient whose complex visual disturbances improved with the maximum dose of 200 milligrams of lamotrigine for six months. Lamotrigine, a commonly prescribed antiepileptic and mood stabilizer, works by decreasing glutamate-mediated excitatory neurotransmission and sodium and voltage-gated calcium channels. Along with that, lamotrigine is widely known for its neuroprotective effect. It is assumed that lamotrigine’s excitotoxic destruction of inhibitory interneurons may be responsible for at least some of the visual symptoms of HPPD [11].

## Conclusions

We suspect this 28-year-old man may have been displaying signs of HPPD despite having one of the more complicated cases with a long history of psychiatric illness and substance abuse. This case demonstrates the vital distinction between HPPD and symptoms associated with schizophrenia, the ineffectiveness of various antipsychotic treatments in HPPD patients, the efficacy of antiepileptic therapy such as lamotrigine, and the validity of the HPPD diagnosis for our patient. However, while keeping in mind the DSM-5 criteria, it is important to note the limitations of this study.
